# All‐Epitaxial Self‐Assembly of Silicon Color Centers Confined Within Sub‐Nanometer Thin Layers Using Ultra‐Low Temperature Epitaxy

**DOI:** 10.1002/adma.202408424

**Published:** 2024-10-12

**Authors:** Johannes Aberl, Enrique Prado Navarrete, Merve Karaman, Diego Haya Enriquez, Christoph Wilflingseder, Andreas Salomon, Daniel Primetzhofer, Markus Andreas Schubert, Giovanni Capellini, Thomas Fromherz, Peter Deák, Péter Udvarhelyi, Song Li, Ádám Gali, Moritz Brehm

**Affiliations:** ^1^ Institute of Semiconductor and Solid State Physics Johannes Kepler University Altenberger Straße 69 Linz 4040 Austria; ^2^ Department of Physics and Astronomy Uppsala University Box 516 Uppsala 75120 Sweden; ^3^ IHP—Leibniz‐Institut für innovative Mikroelektronik Im Technologiepark 25 D‐15236 Frankfurt (Oder) Germany; ^4^ Dipartimento di Scienze Universita Roma Tre Rome 00146 Italy; ^5^ HUN‐REN Wigner Research Centre for Physics P.O. Box 49 Budapest H‐1525 Hungary; ^6^ Beijing Computational Science Research Center Beijing 100193 China; ^7^ Department of Atomic Physics Institute of Physics Budapest University of Technology and Economics Műegyetem rakpart 3. Budapest H‐1111 Hungary; ^8^ MTA‐WFK Lendület “Momentum” Semiconductor Nanostructures Research Group P.O. Box 49 Budapest H‐1525 Hungary

**Keywords:** deterministic position control, epitaxy, quantum light sources, self‐assembly, silicon color centers

## Abstract

Silicon‐based color‐centers (SiCCs) have recently emerged as quantum‐light sources that can be combined with telecom‐range Si Photonics platforms. Unfortunately, using conventional SiCC fabrication schemes, deterministic control over the vertical emitter position is impossible due to the stochastic nature of the required ion‐implantation(s). To overcome this bottleneck toward high‐yield integration, a radically innovative creation method is demonstrated for various SiCCs with excellent optical quality, solely relying on the epitaxial growth of Si and C‐doped Si at atypically‐low temperatures in an ultra‐clean growth environment. These telecom emitters can be confined within sub‐nm thick epilayers embedded within a highly crystalline Si matrix at arbitrary vertical positions. Tuning growth conditions and doping, different well‐known SiCC types can be selectively created, including W‐centers, T‐centers, G‐centers, and, especially, a so far unidentified derivative of the latter, introduced as G′‐center. The zero‐phonon emission from G′‐centers at ≈1300 nm can be conveniently tuned by the C‐concentration, leading to a systematic wavelength shift and linewidth narrowing toward low emitter densities, which makes both, the epitaxy‐based fabrication and the G′‐center particularly promising as integrable Si‐based single‐photon sources and spin‐photon interfaces.

## Graphical abstract

Solid‐state color center emitter formation by ion implantation has the drawback that emitter positions below the surface cannot be deterministically controlled on the nanoscale. Here, this bottleneck can be overcome by a fabrication scheme that departs from ion implantation and only uses epitaxial self‐assembly of color centers. Their formation can be controlled in layers as thin as one lattice constant.

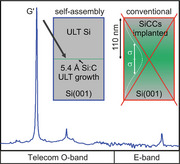

## 1 Introduction

Since the early days of silicon (Si) electronics, the multitude of different Si color centers (SiCCs) induced by ion‐implantation and other treatments in semiconductor technology have been studied extensively.^[^ ^1^ ^]^ The investigations mainly focused on their structural properties, aiming to use SiCCs as fingerprints to probe material quality. However, recent works revealed that isolated, single SiCCs could serve as on‐demand sources of single telecom photons^[^ ^2^, ^3^, ^4^, ^5^, ^6^ ^]^ and light‐matter interfaces^[^ ^7^, ^8^, ^9^ ^]^ – fundamental building blocks for advanced quantum technologies including quantum communication, and computation.^[^ ^10^ ^]^ This huge potential has motivated an ever‐growing scientific community to investigate a variety of well‐known and newly‐discovered optically‐active single SiCCs.^[^ ^4^ ^]^ The most prominent are related to carbon‐(C‐)Si point defects, C‐hydrogen‐(H‐)Si, C‐oxygen‐(O‐)Si complexes, or Si self‐interstitials.^[^ ^1^, ^2^, ^3^, ^4^, ^5^, ^6^, ^7^, ^8^, ^9^, ^11^, ^12^, ^13^, ^14^, ^15^, ^16^, ^17^, ^18^, ^19^, ^20^, ^21^, ^22^ ^]^ For many SiCCs, the research regarding their fundamental structural, electronic,^[^ ^11^ ^]^ and optical properties, as well as benefits and drawbacks of host modifications,^[^ ^21^, ^23^ ^]^ resilience against nearby lattice imperfections is still in its infancy. Despite the lack of yet optimized manufacturing schemes for high‐quality SiCCs, fundamental technological milestones, such as electrically‐pumped emission from SiCC ensembles^[^ ^12^, ^13^, ^14^ ^]^ or their integration into photonic waveguides^[^ ^18^, ^19^ ^]^ and resonators^[^ ^15^, ^16^, ^17^ ^]^ have already been demonstrated. Considering photonic integration, these quantum emitters can significantly benefit from the Si‐based photonic integrated circuit technology,^[^ ^15^, ^16^, ^17^, ^18^, ^19^ ^]^ where they could be monolithically integrated, unlike other proposed solid‐state quantum light sources.

**Figure 1:**
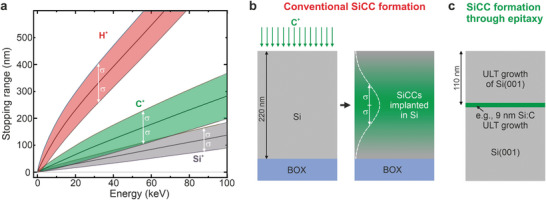
Intrinsic bottleneck of using ion‐implantation for color center formation. a) Simulated stopping range of Si^+^, C^+^ and protons in crystalline Si(001) for different common implantation energies. b) Common fabrication scheme for Si‐color centers (SiCC) consisting of ion‐implantation (here C^+^) and annealing, leading to a stochastic distribution of emitters in the SOI device layer. c) SiCC formation through molecular beam epitaxy growth at ultra‐low sample temperatures (200–300 °C). Efficient confinement of the emitter position down to the nanoscale through the growth of thin Si:C layers on Si bulk or SOI, overgrown with Si at ULT conditions.

Unfortunately, all these defects result from stochastic crystal lattice damage due to ion‐implantation at typical energies ranging from ≈20–150 keV.^[^ ^2^, ^3^, ^4^, ^5^, ^6^, ^7^, ^8^, ^9^, ^11^, ^12^, ^13^ ^]^ This dependency on ion‐implantation poses the major bottleneck toward deterministic high‐yield photonic integration, as implantation leads to extended ion distribution profiles and, thus, insufficient control over the SiCC formation process with typical depth variations of more than hundred nanometers. **Figure** **1**a shows the mean projected range and two times the vertical straggling (σ) obtained from the transport of ions in matter (TRIM) simulations^[^ ^24^ ^]^ for three relevant ion species (Si^+^, C^+^, and H^+^, respectively) implanted into Si. Within a vertical range of at least 100 to 200 nm, the emitter formation occurs stochastically and cannot be controlled, even if only single ions are implanted. Figure 1b depicts a common fabrication scheme for SiCCs on silicon‐on‐insulator (SOI) substrates. The implantation of C^+^ typically leads to the formation of so‐called G‐centers, point defect complexes formed by an interstitial Si atom bound to two substitutional C atoms.^[^ ^1^ ^]^ Typically, high‐energy C^+^‐implantation is followed by thermal annealing and an optional H^+^‐implantation. An implantation energy of ≈35 keV for C^+^ corresponds to a projected range of 110 nm, i.e., the maximum of the defect formation occurs at the center of a 220 nm thick SOI layer and is accompanied by a broad vertical emitter distribution (Figure 1b).

This random formation process limits deterministic photonic and ‐device integration at large scales, which asks for an optimized and reproducible overlap of the vertical emitter position with the field maxima of the photonic modes in waveguides and resonators. Implantation at atypically‐low energies (≤ 5 keV) could minimize the implantation damage to a depth window of just a few nanometers.^[^ ^25^ ^]^ However, the resulting vicinity of the emitter to the surface is non‐advantageous regarding parasitic surface states, poor emitter/mode overlap, and spectral diffusion‐related issues for the indistinguishability of emitted photons. Post‐implantation overgrowth will not be able to eliminate this interface, and the low thermal budget of SiCC during epilayer preparation will always be a major concern. This is particularly true considering the sensitivity of quantum emitters to their local matrix environment.

## 2 Results

### 2.1 Vertical Confinement of Si Color Centers

**Figure 2:**
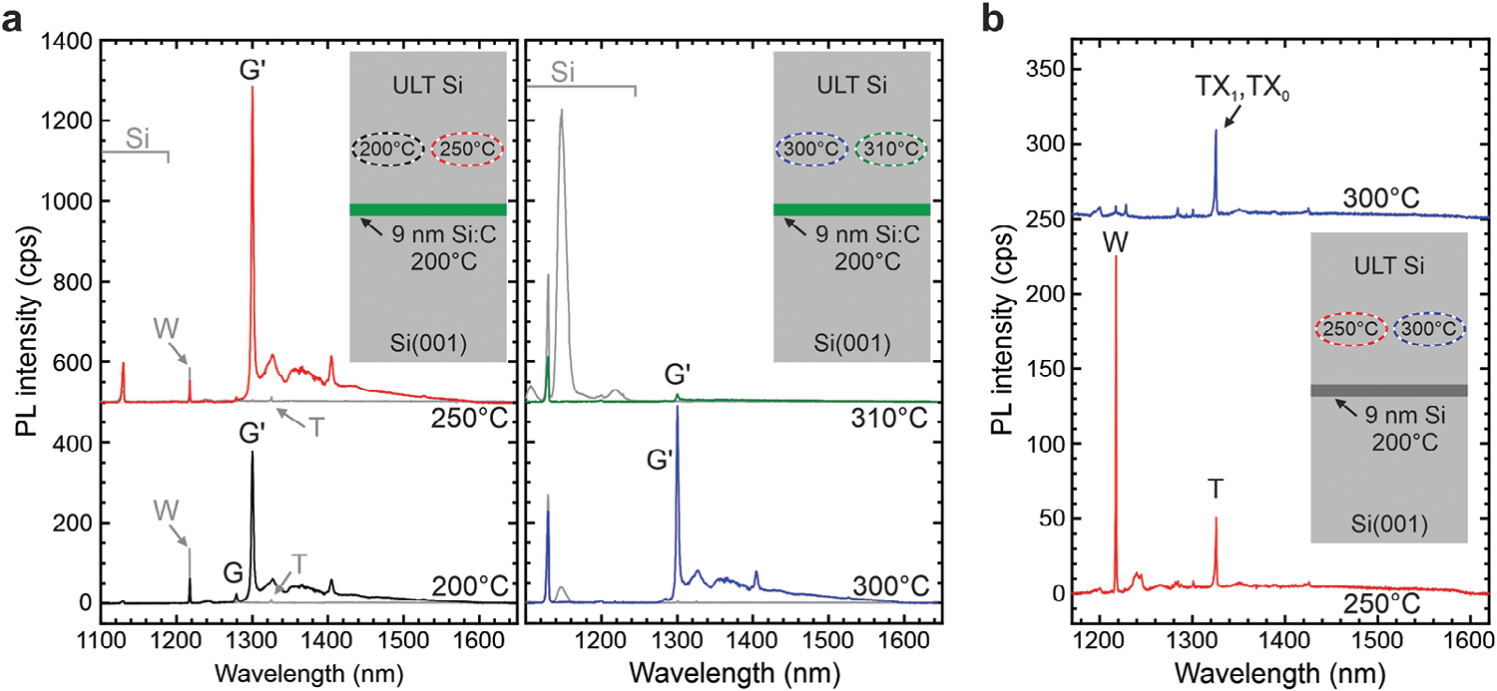
Photoluminescence spectra of Si color centers formed within nanoconfined layers. a) Deposition of 9 nm Si:C at *T*_G_ = 200 °C and overgrown with Si at *T*_cap_ = 200 °C (black spectrum), *T*_cap_ = 250 °C (red spectrum), *T*_cap_ = 300 °C (blue spectrum), and *T*_cap_ = 310 °C (green spectrum). Insets show the respective fabrication scheme. Grey PL spectra originate from pure Si reference samples, grown at the respective *T*_cap_s of 200, 250, 300, and 310 °C. Predominant formation of G′ color centers in the 9 nm Si:C layer and W‐centers in the Si capping layer at *T*_cap_ ≤ 250 °C. b) Predominant self‐assembly of W‐centers (red spectrum) and *T*‐centers (blue spectrum) through the growth of 9 nm Si at *T*_G_ = 200 °C followed by Si overgrowth at *T*_cap_ = 250 °C and *T*_cap_ = 300 °C, respectively. Some spectra are vertically shifted for clarity.

The selective epitaxial creation of SiCCs within sub‐10 nm thick layers and at deterministically chosen depths under the sample surface was first demonstrated. For all applications, the thin SiCC layers must be overgrown to ensure, e.g., optimal emitter/cavity‐mode overlap and provide the necessary vertical separation between emitters and parasitic surface states. A typical growth protocol is depicted in Figure S1 (Supporting Information). A high‐quality Si buffer layer grown at high growth temperature (*T*_G_>500 °C) separates the SiCC‐layer from the initial substrate surface. Then, we grew 9 nm of Si or Si:C at a *T*_G_ = 200 °C. This *T*_G_ was kept constant for Si:C deposition throughout this work. The low *T*_G_ limits the growth kinetics and allows for the epitaxial formation of SiCCs that are subsequently overgrown with Si at capping‐layer growth temperatures *T*_cap_s varying from 200 to 310 °C. We changed *T*_cap_, to find an ideal trade‐off between the Si matrix quality and the thermal budget, which must not negatively affect the SiCC properties, as they collectively annihilate above ≈300 °C.^[^ ^1^ ^]^ In **Figure** **2**, the Si capping layers thickness was ≈105 nm to mimic an emitter position precisely in the middle of a SOI‐220 nm substrate. To unambiguously trace the PL's origin to the SiCC layer, we studied the PL response of pure Si reference layers grown at ULT (see Figure 2).

**Figure 3:**
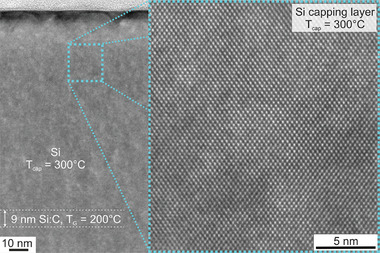
Cross‐sectional transmission electron microscopy. Image of a 9 nm thick Si:C layer (C‐concentration of 3.8 × 10^19^ cm^−3^) capped with crystalline Si grown at *T*_cap_ = 300 °C.

Figure 2a depicts the PL emission from 9 nm thick Si:C layers (C = 3.8×10^19^ cm^−3^) grown at *T*_G _= 200 °C and capped with pure Si at *T*_cap_ = 200, 250, 300, and 310 °C. The superimposed gray spectra in each plot correspond to reference spectra for which the low‐temperature Si:C layer was replaced by a Si layer. For the Si references grown at T_cap _≤ 250 °C, spectrally narrow emission at 1220 nm and 1325 nm is observed (grey arrows), corresponding to W‐center and T‐center zero phonon lines (ZPL).^[^ ^1^ ^]^ For T_cap _≥ 300 °C, the PL spectra of the reference samples in Figure 2a indicate an excellent crystal quality, as evidenced by two findings. First, for *T*_cap_ = 300 °C, only faint PL emission lines below the Si bandgap are visible that vanish at *T*_cap_ = 310 °C. Second, at *T*_cap_ ≥ 300 °C, we also observe a broad electron‐hole droplet (EHD) related emission from the Si matrix layer at a wavelength of 1150 nm, a sign of high crystal quality and low density of non‐radiative recombination channels. Note that for T_cap _≤ 250 °C, for which W‐ and T‐center PL is observed in the reference samples, no EHD emission from Si exists. We also note that for the samples containing the Si:C layer, no EHD emission is observed, as carriers efficiently recombine within the SiCC layer. The excellent crystalline quality of the Si capping layer at *T*_cap_ = 300 °C is confirmed by high‐resolution cross‐sectional TEM, see **Figure** **3**.

In Figure 2a, the shapes of the grey reference PL signals starkly contrast the signal from the samples containing the C‐doped layer. For all C‐doped samples, we observe a prominent ZPL line at 1299.8 nm (953.8 meV). We note that the observed ZPL wavelength deviates from that of a G‐center at –1278 nm (Ref. [1]), but its observed Debye–Waller factor of 17.9% is very similar to that reported for G‐center ensembles.^[^ ^4^ ^]^ Like the G‐center, the here‐found emission center consists of a ZPL, a broad phonon side band at higher wavelengths, and a local phonon mode (LPM) that is shifted by about –71 meV to lower energies (Figure S3a, Supporting Information). Due to the similarities in their spectral shape, we label the emitter as G′. Indeed, the here‐found G′‐center might be one of the G‐center‐related lines found in an earlier work by Davies et al. (Ref. [26]). Detailed ab initio calculations of the G′‐centers atomic configuration and optical properties will be given in Section 2.3. below.

For the SiCC layer with *T*_cap_ ≤ 250 °C, the W‐center emission is weaker than for the corresponding reference sample (*T*_cap _≤ 250 °C), pointing to a dominant carrier capture within the thin Si:C layer. At *T*_cap_ = 300 °C, blue spectrum in Figure 2a, we still observe a strong G′center signal, while the background W‐center signal from the Si matrix vanished.

This finding strongly indicates that the SiCC emission is unambiguously dominantly originating from the thin ULT Si:C region. Segregation of C at *T*_G_s of Si:C nanolayer and Si capping layer can be excluded.^[^ ^27^ ^]^ The ratio of the integrated G′‐ZPL intensity of the sample with C = 3.8 × 10^19 ^cm^−3^ and *T*_cap_ = 300 °C relative to the reference sample without C‐deposition is as high as 310:1. However, we note that the thermal budget of the SiCCs is critical since the thermal annealing induced by overgrowth at a *T*_cap_ = 310 °C induces a strong quenching of the G′‐center emission (green spectrum in Figure 2a).

Results of excited state lifetime measurements obtained by time‐correlated single photon counting for ensembles of G′‐centers, overgrown at *T*_cap _= 200 °C and 300 °C, are shown in Figure S3b (Supporting Information). The PL lifetimes are ≈7 ns for both samples, a value close to the one observed for G‐centers,^[^ ^2^, ^6^, ^18^ ^]^ and significantly shorter as compared to the lifetimes observed for W‐ and T‐centers.^[^ ^5^, ^7^ ^]^

Nevertheless, the occurrence of W‐ and T‐centers in the Si reference samples grown at *T*_cap _≤ 250 °C offers the further possibility to select the emitter type within a nanolayer. By growing a 9 nm thick Si layer at *T*_G_ = 200 °C and overgrowing it with Si at *T*_cap_ = 250 °C and *T*_cap_ = 300 °C, respectively, W‐centers and T‐centers^[^ ^1^ ^]^ can be predominantly grown in the nanolayer, Figure 2b. The W‐centers created recent interest as single‐photon emitting SiCCs,^[^ ^5^ ^]^ while the T‐center, a Si‐C‐H point defect complex, is particularly promising due to its long spin lifetimes and spin‐selective optical transitions.^[^ ^21^ ^]^

### 2.2 Influence of C‐Doping and Layer Thickness on SiCC Emission

**Figure 4:**
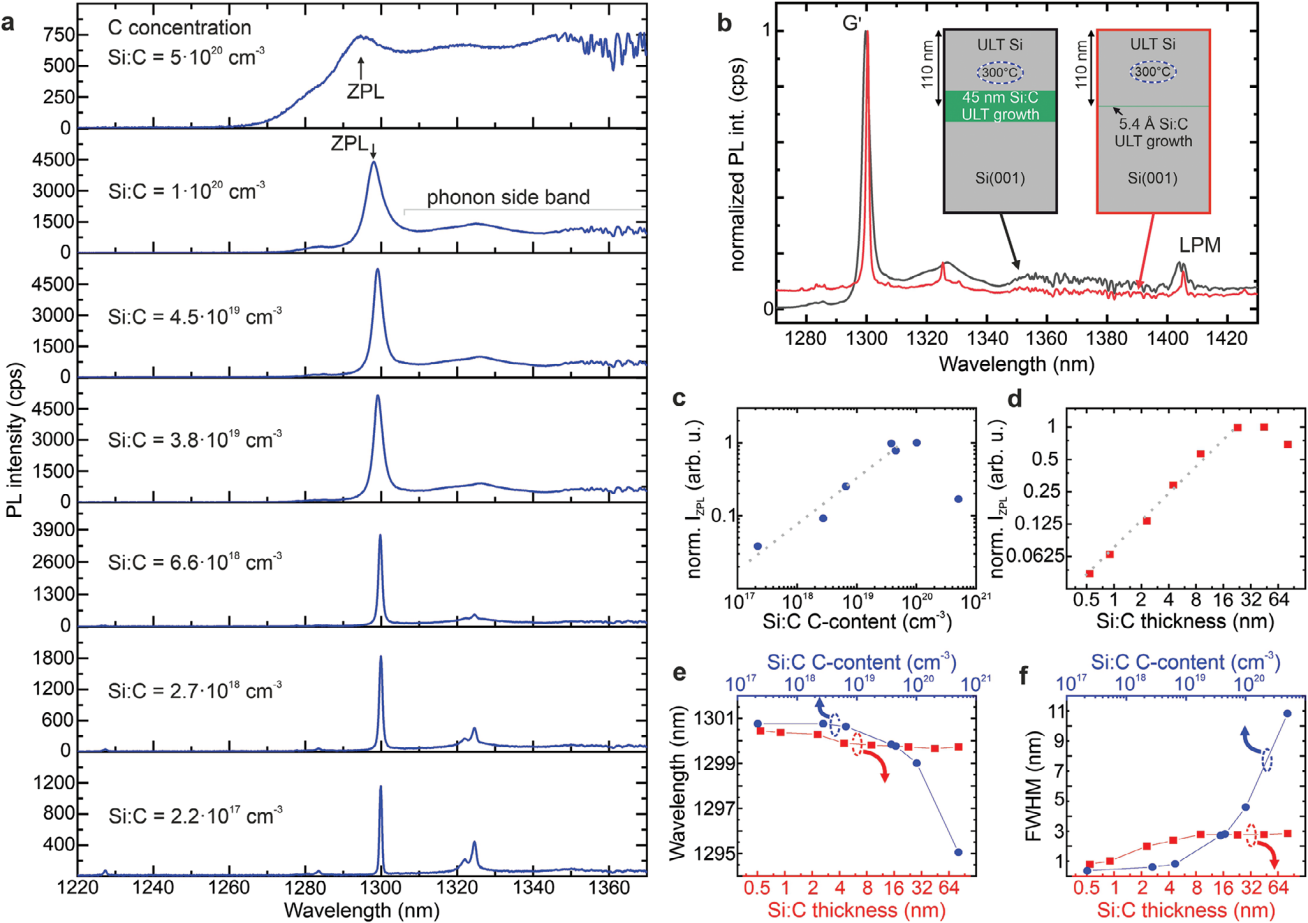
Photoluminescence spectra of Si color centers. a) SiCCS, formed upon deposition of a 9 nm thick C‐doped Si layer with various C concentrations, deposited at *T*_G_ = 200 °C and overgrown at *T*_cap_ = 300 °C. b) Si:C layers of 45 nm thickness (black spectrum) and 0.5 nm thickness (red spectrum), deposited at *T*_G_ = 200 °C and capped at *T*_cap_ = 300 °C. A C‐concentration of 3.8 × 10^19 ^cm^−3^ was used. Insets show a scheme of the respective sample structure. c) Influence of the C‐concentration in the Si:C layer on the normalized integrated PL intensity of the ZPL (I_ZPL_). d) Influence of the Si:C layer thickness on I_ZPL_. Dotted grey lines in c and d are guides to the eye. e) Influence of the Si:C layer thickness and C‐concentration in the Si:C layer on the emission wavelength of the zero‐phonon line of the G′ center. f) Influence of the Si:C layer thickness and C‐concentration in the Si:C layer on the (FWHM) of the zero‐phonon line of the G′ center.

Previously, we focused on a high C‐concentration (3.8 × 10^19 ^cm^−3^) and a Si:C layer thickness of 9 nm to determine the SiCC's vertical selectivity. Next, we focus on the influence of the C‐concentration and the Si:C layer thickness on the PL emission. **Figure** **4**a depicts the impact of a decreasing C concentration (C = 5 × 10^20^cm^−3^ to 2.2 × 10^17^cm^−3^) in a 9 nm thick Si:C film that was subsequently capped with ≈105 nm of Si, grown at *T*_cap _= 300 °C. For low C‐concentrations of 2.2 × 10^17 ^cm^−3^, the spectrum is dominated by the ZPL of the G′‐center at 1300.8 nm (953.2 meV) (Figure 4a,e,f) with a detectable full width at half maximum (FWHM) of less than 0.4 nm (<300 µeV). With increasing C‐concentration, the PL intensity of the G′‐ZPL emission increases (Figure 4a,c). At the same time, the ZPL emission blue‐shifts (Figure 4a,e), and the FWHM increases, see Figure 4f. The extension of the electronic wave function associated with the G′ ZPL transition is supposed to be small compared to the expected mean G′ to G′ distance. Thus, we suggest that the observed inhomogeneous broadening of the ZPL and its blue shift for higher C contents is associated with local strain fields caused by non‐radiative C‐related defects within the surrounding matrix. For very high C‐concentration of 5×10^20 ^cm^−3^, the spectrum is dominated by the emission of the phonon‐related sideband, exhibiting only a broad zero phonon peak at about 1295 nm (Figure 4a,f). At the lowest investigated C‐concentrations, the spectral signature of T‐centers is observed at wavelengths around 1325 nm (TX_0_ at 1326 nm and TX_1_ at 1323.5 nm).^[^ ^8^ ^]^

Figure 4b depicts the differences in PL emission from SiCC layers with thickness of 45 nm and 5.4 Å (0.54 nm). Both spectra are normalized to the maximum of their G′‐ZPL emission. The comparison indicates a significant narrowing of the ZPL for a strongly reduced Si:C layer thickness. The inset in Figure 4b shows the respective sample layout, with both Si:C layers being doped with C to a concentration of 3.8 × 10^19 ^cm^−3^. The capping layer thickness (*T*_cap _= 300 °C) was adjusted so that the middle of the Si:C layer was exactly 110 nm below the sample surface. The dependence of the ZPL intensity, transition wavelength, and linewidth on the Si:C layer thickness is shown more systematically in Figure 4d–f. Continuously increasing the Si:C layer thickness from 0.5 nm to ≈30 nm, the PL intensity of the G′‐ZPL increases linearly with increasing film thickness (Figure 4d), pointing to an excellent scalability of the SiCC densities. For larger thickness, (≥45 nm), the emission intensity saturates and even slightly decreases. We assign this decrease to the thinner Si capping layer for thicker Si:C layers and, thus to a decreased in situ annealing time, associated with the Si capping layer growth at *T*_cap_ = 300 °C. Comparing the emission intensities in Figure 2a, it is evident that moderate annealing at 250–300 °C increases the emission intensity from SiCCs. At the same time, the emission wavelength of the G′‐ZPL slightly increases with decreasing thickness (Figure 4e), while the FWHM of the G′‐ZPL decreases. In line with our interpretation of the respective trends observed for variable C content, significant changes just occur for thin Si:C layers as the strain field becomes quasi 2D. Thus, its variance is reduced compared to the 3D inhomogeneous strain fields in thick C‐doped layers.

The characteristics of these interstitial‐related SiCCs significantly constrain the process parameter window for in‐growth defect engineering. The relatively‐low thermal budget that can be tolerated by SiCCs before annihilation imposes the use of ULT epitaxy, with growth temperatures (*T*_G_ and *T*_Cap_) limited to <300 °C. Earlier works in proton‐implanted samples found that for traditional G‐centers, disintegration starts already at ≈175 °C for a 30 min annealing step in an argon atmosphere, while for W‐centers, this temperature is ≈270 °C.^[^ ^28^ ^]^ Therefore, a suitable *T*_G_‐balance between SiCC generation and preservation or emitter reduction during Si capping and annealing has to be considered. Here, we find that growing SiCCs at *T*_G _= 200 °C and embedding them in Si grown at *T*_cap _= 300 °C minimizes the damage in the Si matrix. A finer adjustment of T_G_s will likely lead to further optimization of the emitter/matrix quality. We also emphasize the need for ultra‐low chamber pressures *during growth*. At growth temperatures < 350 °C, residual gases are not efficiently desorbed from the Si surface,^[^ ^29^ ^]^ i.e., foreign residual atoms that constitute the growth chamber pressure (background plus pressure increase due to hot sources) are also incorporated. The relation between growth pressure and the rate of residual gas impingement^[^ ^30^ ^]^ is shown in Figure S1 (Supporting Information). These foreign atoms can form detrimental defects that, in the worst case, disturb SiCC emission through the generation of (non)‐radiative recombination channels and provide an unfavorably noisy matrix environment for quantum emitters. Here, we find a spurious PL response from T‐centers (C‐H‐related defects), despite excellent growth pressures in the low 10^−10^ mbar range (see Figure S1, Supporting Information). However, this finding guides future experiments for the creation of high‐quality isolated T‐centers. We note that many different point‐defect centers are known to exist in Si.^[^ ^1^, ^2^, ^3^, ^4^, ^5^, ^6^, ^7^, ^8^, ^9^, ^11^, ^12^, ^13^, ^14^, ^15^, ^16^, ^17^, ^18^, ^19^, ^20^, ^21^, ^22^ ^]^ However, due to the novelty of their application potential in quantum communication, it can be expected that only a fraction of defect‐centers with optimized quantum properties are known to date. Their properties will depend on factors like, e.g., types of foreign atoms at the point defect site, strain, matrix composition, etc.

**Figure 5:**
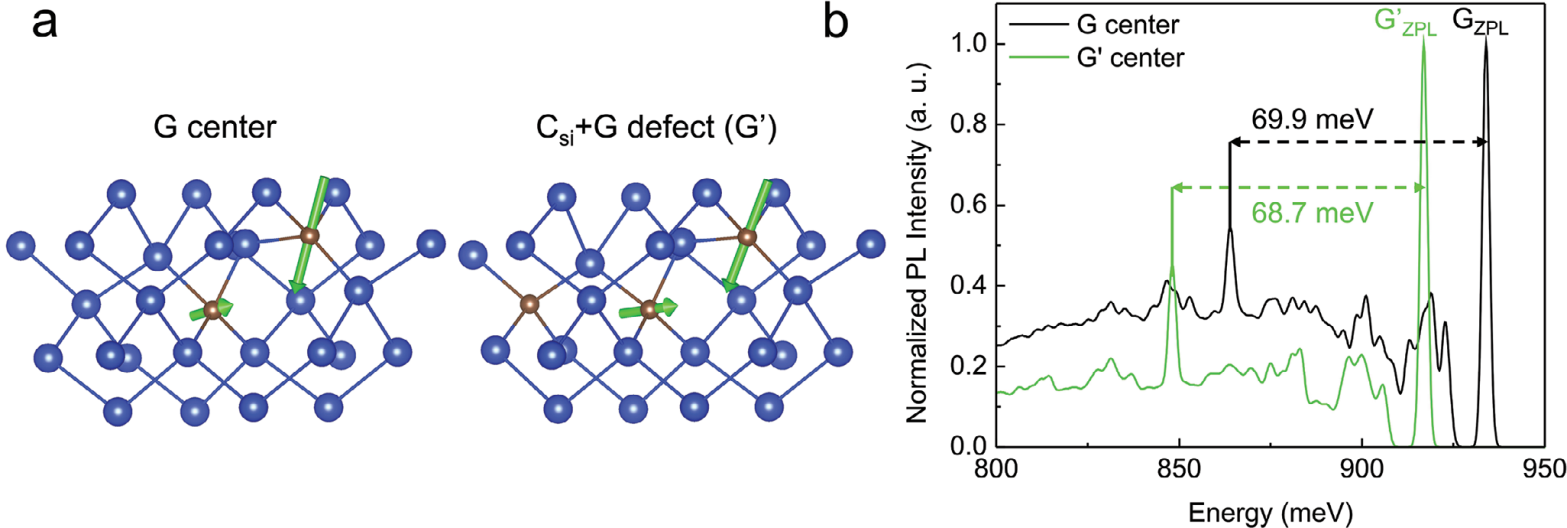
Model for the G′‐defect and calculated luminescence emission. Prominent local vibration mode in the G‐center and C_Si_+G defect (G′‐center). a) The geometry of the defects is depicted where the black circle indicates the location of C_Si_. The arrows show the direction and the amplitude of vibrating ions that are mainly localized on the two carbon atoms near the Si interstitial. b) The simulated PL spectrum of the G‐center and C_Si_+G defect (G′‐center) with the prominent local vibration modes as depicted in (a).

Here, we demonstrate the epitaxial creation of various vertically confined emitters, such as G, G′, W‐, and T‐centers that are, when isolated, promising candidates for quantum photonic applications. In particular, our ab initio calculations show (see **Figure** **5** and Supporting Information) that G′‐center should exhibit a similar optically detected magnetic resonance (ODMR) signal as its G‐center counterpart.^[^ ^31^ ^]^ Thus, the G′‐centers have a great potential to act as a quantum memory together with emission in the telecom O‐band.

### 2.3 Ab initio Calculations of the G′‐Center

We employed ab initio calculations to identify the G′‐center and reveal its potential for quantum technologies. We assumed, based on the similarities between the features of the PL spectra of the G‐center and G′‐center, that the core of the defect is the atomic structure of the G‐center but a nearby C impurity or Si self‐interstitial may perturb it. The possible models employed were C substitutional (C_Si_), C interstitial (C_i_) or silicon interstitial (Si_i_) near C_Si_‐Si_i_‐C_Si_ defect (so‐called B‐form of G‐center that we simply label by “G” for the sake of simplicity) that are depicted in Figure S4 (Supporting Information).

Our ab initio calculations imply that the G′‐center consists of three C atoms where a substitutional C sits next to the atomic configuration of the G‐center which well reproduces the characteristic features of the PL spectra: red‐shift both in the ZPL and the prominent LPM near the ZPL (also‐called “E” peak^[^ ^1^ ^]^) in the G′‐center when compared to the G‐center (see Figure 5b). The observed (calculated) red‐shifts in the ZPL energies of G′ versus G are 17 meV (14 meV), whereas the energy of the E‐peak with respect to the corresponding ZPL differs by 0.8 meV (1.2 meV) in the experimental (simulated) spectra. We note that our C‐source i.a. emits molecules with three C atoms that might be built in as a unit to the Si crystal, which may facilitate the dominant formation of the G′‐center.

We also find that the G′‐center has a triplet level at 0.63 eV above the ground state (Figure S5, Supporting Information). The position of this metastable triplet level is reminiscent to the position of the metastable triplet level in the G‐center (0.67 eV). In our previous work,^[^ ^11^ ^]^ we showed that this metastable triplet state was observed in the optically detected magnetic resonance (ODMR) of the G‐center. Since the level structures of G‐center (Ref. [11]) and G′‐center are very similar it is highly plausible that G′‐center experiences the same ODMR effect. While the exact microscopic mechanism behind the ODMR effect for the G‐center has not yet been revealed, the occurrence of the ODMR signal for the G‐center clearly indicates that optical spin‐polarization and optical readout of the electron spin in the metastable triplet state are doable. We propose that the same mechanism should occur in the G′‐center too, so the same optical spin‐polarization and readout techniques can be applied for the G′‐center. We argue that our experiments realize precise control of the location and density of the G′‐center, which has become a very promising candidate for realizing spin‐to‐photon interface with emission in the telecom O‐band.

## 3 Discussion

This first demonstration of purely epitaxial growth of self‐assembled SiCCs marks the critical initial step toward deterministic vertical position control in the nanometer range. As a proof of concept, we have confined the G′ emitters within an only one unit cell thick layer (5.4 Å). Additionally, omitting the overall damaging impact of ion‐implantation promises emitter generation within a matrix material that can be electronically more “quiet”, important for any kind of quantum application, especially considering the detrimental influence of spectral diffusion on the indistinguishability of quantum emitters.^[^ ^32^ ^]^ In this work, these ground‐breaking methods have already been shown to be excellent for forming different SiCC types, and a successful extension to even more types can be expected. We point out that this initial work was targeted toward a proof‐of‐principle and a vast parameter space for optimizing the SiCCs with respect to their quantum optical benchmarks remains untapped.

As the next milestone to achieve laterally separated single SiCCs, a systematic reduction of C‐concentrations, layer thicknesses, and annealing or a combination of these has to be addressed. Sample annealing to dilute the emitter density, performed in situ in the MBE's UHV environment after Si matrix growth is a logical next step. Furthermore, conventional ex situ annealing methods as employed for ion‐implanted SiCCs can be used. Additionally, templated local epitaxy schemes for fabricating self‐assembled SiCCs can be envisioned to control the lateral emitter position. Until then, deterministic processing techniques that have been developed for randomly nucleated group‐III‐V QDs, like in situ optical lithography,^[^ ^33^ ^]^ cathodoluminescence lithography,^[^ ^34^ ^]^ and two‐color PL imaging^[^ ^35^, ^36^ ^]^ can be applied to self‐assembled SiCCs.

The use of in situ defect incorporation and growth of the host Si crystal has several further advantages, providing various opportunities. It is viable to grow Si on top of SOI. Therefore, all resonator structures (microdisks, photonic crystals, bulleyes, waveguides, etc.) can be conveniently fabricated top‐down. In contrast to other approaches,^[^ ^37^ ^]^ our present method can straightforwardly position SiCCs deterministically at vertical positions, where the photonic mode has a maximum. Based on previous results, we envision that Q‐factors of 10^5^–10^6^ can be achieved in, e.g., photonic crystal cavities.^[^ ^38^, ^39^ ^]^ This is definitely of great importance for *T*‐centers for which the brightness and the quantum yield is not sufficiently high for pursuing quantum optical protocols on single defects.^[^ ^7^ ^]^ In addition, we envision deterministic strain control for SiCCs with our scheme. Strain control on SiCCs is of prime importance as the spin levels^[^ ^7^, ^40^ ^]^ and the position and polarization of the ZPL emission are greatly sensitive to the strain field in Si.^[^ ^41^ ^]^ By adding Ge to the Si crystal, Si_1‐x_Ge_x_ heterostructures can be readily grown where the variation of composition *x* can be used to tune the strength of the desired strain field. Indeed, our ULT‐MBE growth applied to SiGe heteroepitaxy^[^ ^42^ ^]^ also allows for the creation of high‐quality planar top‐down platforms for advanced nanoelectronics devices.^[^ ^43^, ^44^ ^]^ Furthermore, the proposed growth technique can be employed to realize long coherence times for the electronic spins of SiCC qubits and engineer the ancilla qubits around the SiCC qubits such as T‐center. We note that once the ODMR signal from a single G‐center or G′‐center is observed, it could step in as a strong contender in the field of quantum communication, where nuclear spin quantum memories are essential in realizing quantum repeaters. By using ^28^Si enriched growth of SiCC layer and its immediate surroundings, the coherence times of SiCC may reach milliseconds^[^ ^8^, ^45^ ^]^ where the nuclear registers can be engineered at the target distance by adding natural Si or any other artificial isotope contribution by design. This can be used to tune the coupling of the electron spin and nuclear spin so as to increase the rate of quantum information processing between the nuclear spin register and the electron spin. Furthermore, the quasi‐2D layer of *I *= 1/2 nuclear spins can be used in quantum simulation protocols^[^ ^46^ ^]^ or to build up a quantum chip when the individual nuclear spins can be addressed and manipulated with the combination of microwave and radiofrequency pulses.^[^ ^47^ ^]^ Finally, vertical *p–i–n* junctions can be readily fabricated by this growth technique^[^ ^48^ ^]^ where the SiCCs are engineered in the intrinsic region to avoid charge fluctuations under illumination of SiCCs^[^ ^49^ ^]^ and apply controllable Stark‐shift on the SiCC's ZPL or to realize photoelectric readout of spins.^[^ ^50^, ^51^, ^52^, ^53^ ^]^

## 4 Conclusion

In summary, we expect that the presented ULT‐growth approach for creating vertically controlled SiCCs can be a starting point for exploring the untapped potential of telecom SiCC quantum emitters and qubits with highly homogeneous environment because of the non‐invasive creation of SiCCs and the full control of optical, electrical, strain and spin environments around SiCCs. Furthermore, this fully integrated quantum electro–optics device can be readily connected to optical fibers by tapering^[^ ^54^ ^]^ to the silicon nanobeams or nanopillars in order to maximize the photon output and integrate this into commercial optics devices.

## 5 Experimental Section

### Epitaxial Growth

All samples were grown on 17.5 mm × 17.5 mm float zone (FZ) Si(001) substrates (*R*_0_ > 5000 Ωcm) cut from a 4 inch wafer. Subsequently, the protective photoresist applied for cutting was removed within a precleaning procedure based on solvents (acetone and methanol) and a UV–ozone surface treatment. Before the growth, the substrates were prepared using a Radio Cooperation of America (RCA) cleaning and were dipped in diluted hydrofluoric acid (HF1%) to remove the native oxide before being introduced into the load lock chamber. All samples were degassed at 700 °C for 15 min, followed by a conditioning step at 450 °C for 30 min prior to the growth. A 75.5 nm thick Si buffer layer was grown at a *T*_G_, ramped from 650 to 600 °C. For all samples containing a layer of C‐doped Si, *T*_G_ was ramped down during a growth interrupt to 200 °C, the growth temperatures of all SiCCs (see Figure S1, Supporting Information). To investigate the *T*_cap_ dependence, 9 nm thick Si:C layers with a nominal Si growth rate of 0.5 Å s^−1^ and C‐concentrations of 3.8 × 10^19^ cm^−3^ at *T*_G_ = 200 °C were deposited. Hereafter, the substrate temperature was ramped to the respective Si *T*_cap_ of 310, 300, 250 or 200 °C, and a 104.5 nm thick Si capping layer was grown at a growth rate of 0.75 Å s^−1^. The respective Si capping layers were deposited directly onto the Si buffer layers for the reference samples. For the investigation of the C‐concentration dependence, the Si:C layers' thickness was kept constant at 9 nm, while the C concentration varied from 2.2 × 10^17^ cm^−3^ to 5.0 × 10^20^ cm^−3^. The carbon deposition rates were calibrated using secondary‐ion mass spectrometry (SIMS) experiments of calibration layers. For the investigation of the Si:C layer thickness dependence, the C‐concentration to 3.8 × 10^19^ cm^−3^ was fixed while the thickness of the Si:C layers was set to 0.54, 0.9, 2.3, 4.5, 9, 23, 45, and 82 nm. The buffer and cap thicknesses were adapted accordingly to maintain the same overall epilayer thickness.

### Photoluminescence Characterization

To perform micro‐photoluminescence (µ‐PL) measurements at low temperatures (5 K), the samples were glued to the coldfinger of a liquid‐helium (LHe) flow cryostat. For excitation, a continuous‐wave (cw) diode‐pumped solid‐state (DPSS) laser emitting at 473 nm and a laser power of max. 6 mW (measured below the cryostat window) were used. The laser was focused, and the luminescence signal was collected via an infinity‐corrected microscope objective with 0.26 numerical aperture (NA) for the ensemble measurements. The µ‐PL spectra were recorded via a 500 mm focal‐length Czerny–Turner spectrometer with three interchangeable ruled gratings (100, 300, and 900 mm^−1^) connected to a liquid‐nitrogen (LN_2_) cooled 1024 pixel InGaAs line detector.

### Transmission Electron Microscopy

Preparation for TEM was done conventionally by grinding, polishing, and Ar ion thinning. A FEI Tecnai Osiris operated at an acceleration voltage of 200 kV for TEM investigation was used.

### Ab Initio Calculation Methods

The considered defects were modeled by supercell plane wave density functional theory (DFT) as implemented in VASP.^[^ ^55^, ^56^, ^57^ ^]^ A 512‐atom (4 × 4 × 4 multiple of the conventional Bravais‐cell) was used, and all atoms were allowed to relax in a constant volume till the forces were below 0.01 eV Å^−1^. The Γ‐point approximation was used for Brillouin‐zone sampling. The lattice constant was taken from our earlier Heyd–Scuzeria–Ernzerhof HSE06 work to be 5.4307 Å (in good agreement with the experiment.^[^ ^58^ ^]^ The zero phonon line (ZPL) was obtained as the energy difference of the relaxed ground and excited states, the latter calculated with constrained occupation or ΔSCF method. To obtain the correct ZPL for the singlet‐to‐singlet transition, an exchange correction was applied (e.g., Ref. 11). The spectrum of the phonon replicas was computed by the generating function method,^[^ ^59^ ^]^ based on vibration calculations using the Perdew‐Burke‐Ernzerhof (PBE) functional.^[^ ^60^ ^]^

## Conflict of Interest

The authors declare no conflict of interest.

## Supporting information



Supporting Information

## Data Availability

The data that support the findings of this study are available from the corresponding author upon reasonable request.
