# Supplementary material for: All‐Epitaxial Self‐Assembly of Silicon Color Centers Confined Within Sub‐Nanometer Thin Layers Using Ultra‐Low Temperature Epitaxy
Source: Adv Mater. 2024 Oct 12;36(48):2408424. doi: 10.1002/adma.202408424 (PMC11602677; doi:10.1002/adma.202408424)
Supplement: Supplementary file 1 — Supporting Information [file ADMA-36-2408424-s001.docx]

Supporting Information

All-Epitaxial Self-Assembly of Silicon Color Centers Confined Within Sub-Nanometer Thin Layers Using Ultra-Low Temperature Epitaxy

Johannes Aberl, Enrique Prado Navarrete, Merve Karaman, Diego Haya Enriquez, Christoph Wilflingseder, Andreas Salomon, Daniel Primetzhofer, Markus Andreas Schubert, Giovanni Capellini, Thomas Fromherz, Peter Deák, Péter Udvarhelyi, Li Song, Ádám Gali, and Moritz Brehm

**Ultra-low temperature epitaxy**

Conventional wisdom suggests that epitaxy growth temperatures (T_G_) above ~500°C are needed to keep point defect densities low and the epitaxial quality high. However, at typical silicon (Si) T_G_s of 500°C-700°C, Si-based quantum emitters, such as G-centers or W-centers, disintegrate within common growth times by recrystallization.^1,2^ Maintaining the SiCC emitter properties requires reducing the T_G_s far below conventional values, i.e., ULT growth as Si quantum emitters like G-centers could sustain a thermal budget of T_G_s ~300°C for several tens of minutes.^1,2^ Therefore, playing with the thermal budgets for Si color center (SiCC) formation and annihilation, ULT growth can, in principle, allow for the formation of SiCCs due to kinetic limitations while allowing for overgrowth with high-quality Si (see Fig. 2).

**Importance of the growth pressure**

Typically, for molecular beam epitaxy (MBE) growth, a growth pressure (P_G_) of 10^-8^-10^-9^ mbar (at base pressures around 1∙10^-10^ mbar) has been considered sufficient but leads to a rate of gas impingement on the substrate of 10^-2^ - 10^-3^ monolayers (ML) per second, see inset of **Figure S1**a.^3^ But at high T_G_, the sticking coefficients are small, and the gas can desorb from the Si surface. ^3^ However, ULT growth is less forgiving, as impurities *will* stick to the layer. ^4^ Thus, at a growth rate of 0.1 Å/s, one impurity per 10-100 Si or Ge atoms could be incorporated. Increasing the growth rate can be beneficial, but clearly, the main knob to turn is reducing P_G_ to a minimum. To keep these impurities at "intrinsic semiconductor" levels, i.e., below 10^16^ cm^-3^, less than one foreign atom per 10^6^ crystal atoms must be incorporated. To achieve these conditions, we have applied extensive and prolonged chamber conditioning, degassing, pumping, and gettering strategies to reduce P_G_ during MBE growth deep into the ultra-high-vacuum range (see Figure S1). For our growth rates, a ratio of growth rate vs rate of gas impingement reaches 10^5^ to 10^6^ or higher for P_G_ ≤ 5∙10^-11^ mbar, see Figure S1. Even for highly C-doped Si layers for which the C source is heated up to >1500°C, P_G_ remains at ~10^-10^ mbar, see Figure S1b.


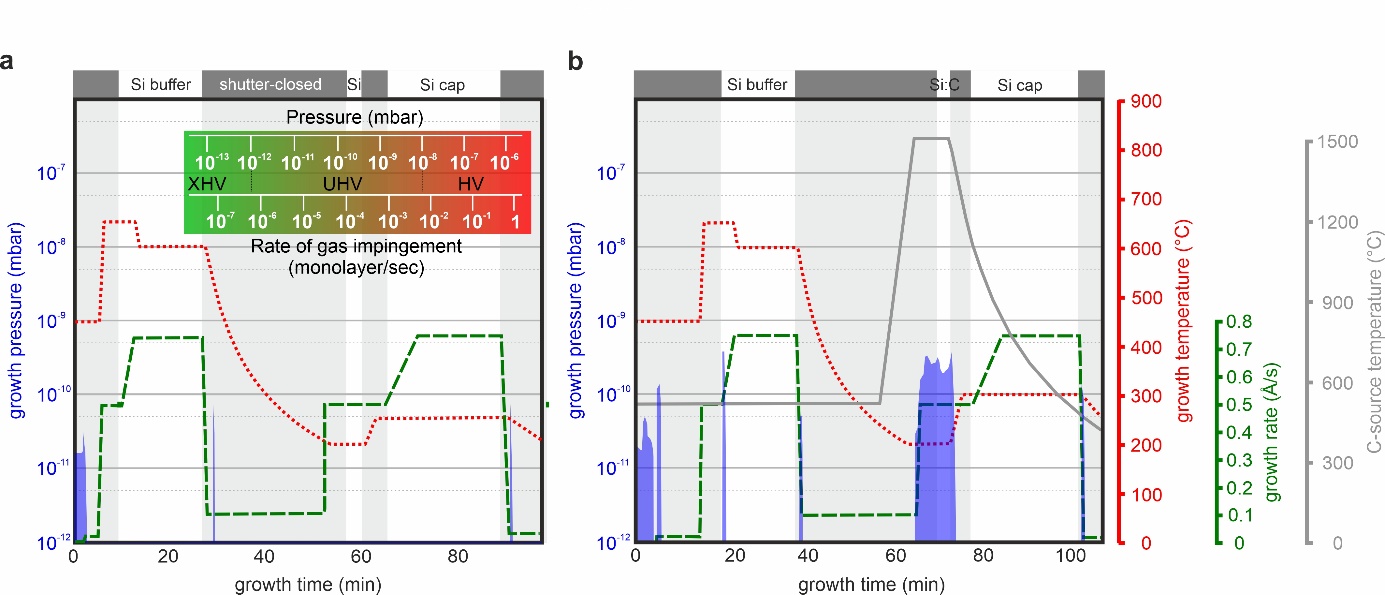


**Figure S1.** *Epitaxial growth conditions.* Untypically-low growth pressures are needed for ULT growth of deterministic layer quality. Growth parameters vs. growth time: Growth pressure (blue areas), substrate temperature (red dotted lines), Si growth rate (green dashed lines), and carbon source temperature (grey curve). **a** Growth parameters for W-center formation within a 9 nm thin layer, grown at 200°C and overgrown at 250°C. **b** Growth parameters for G′-center formation within a 9 nm thin Si layer doped with C (3.8∙10^19^ cm^-3^). Inset in **a**: Relationship between growth pressure and rage of residual gas impingement, essential for epitaxial growth at temperatures <350°C. Within the gray shaded areas, the growth temperature was ramped to its setpoint with all effusion cells and evaporators closed.

**MBE versus CVD**

For forming self-assembled color centers, epitaxy methods like MBE are necessary. The standard in Si-related industries is chemical vapor deposition (CVD), also because many wafers can be overgrown in parallel by CVD. MBE, however, is versatile and exceptionally flexible, and many advances in semiconductor technology have thus been based on MBE. The particular key feature of MBE is the complete decoupling of T_G_ and the growth rates of atoms impinging on the substrate. This is not the case for CVD, which requires the chemical decomposition of precursor gases that only occurs at elevated T_G_s. For the creation of Si-based color centers, the game changer in favor of MBE is the accessibility of the ULT regime (T_G_ = 100°C - 300°C). For CVD, decreasing T_G_ for epitaxy results in a decrease in the growth rate until the processes become ineffective. Regular precursors only provide reasonable growth rates of a few nm/min down to T_G_∼500°C for SiH_4_ and ∼350°C for GeH_4_.^5^ For highly epitaxial Si layers, necessary growth temperatures of ≤300°C cannot be reached by epitaxy methods that rely on the decomposition of precursor gases.

We note that MBE is used in industry, e.g., for the epitaxial growth of vertical cavity surface emitting lasers (VCSELs), and coupled CVD - MBE systems are employed to grow III-V quantum dot lasers on Si for Si photonics purposes. Thereby, the relaxed buffers are grown by CVD and the active emitters by MBE. Following the needs of the industry, a high-capacity MBE production tool (MBE 8000), able to handle four 200 mm wafers, has become recently available through a joint development program of Riber SA and IntelliEPI.^6^

**TEM analysis of the Si capping layer**

From the photoluminescence (PL) spectra presented in **Figure 2** of the main text, we found that a T_cap_ of 300°C minimizes the signal from here unwanted point defect emission centers like W- or T-centers while maintaining the emission intensity of the underlying G′-center layer. Here, we additionally investigated the structural quality of the Si capping layer grown at T_cap_ = 300°C by high-resolution cross-sectional transmission electron microscopy. Preparation for TEM was done conventionally by grinding, polishing, and Ar ion thinning. We have used a FEI Tecnai Osiris operated at an acceleration voltage of 200 kV for TEM investigation.
Figure S2 presents the overview image and a high-resolution section. The contrast between the high-temperature Si buffer, the low-temperature Si:C (9 nm thickness and C-concentration of 3.8∙10^19^ cm^‑3^), and the Si capping layer is too weak to see clear interfaces. The high-resolution image on the right side of Figure S2, highlights the excellent crystalline quality of the Si capping layer, despite the low growth temperature of 300°C.


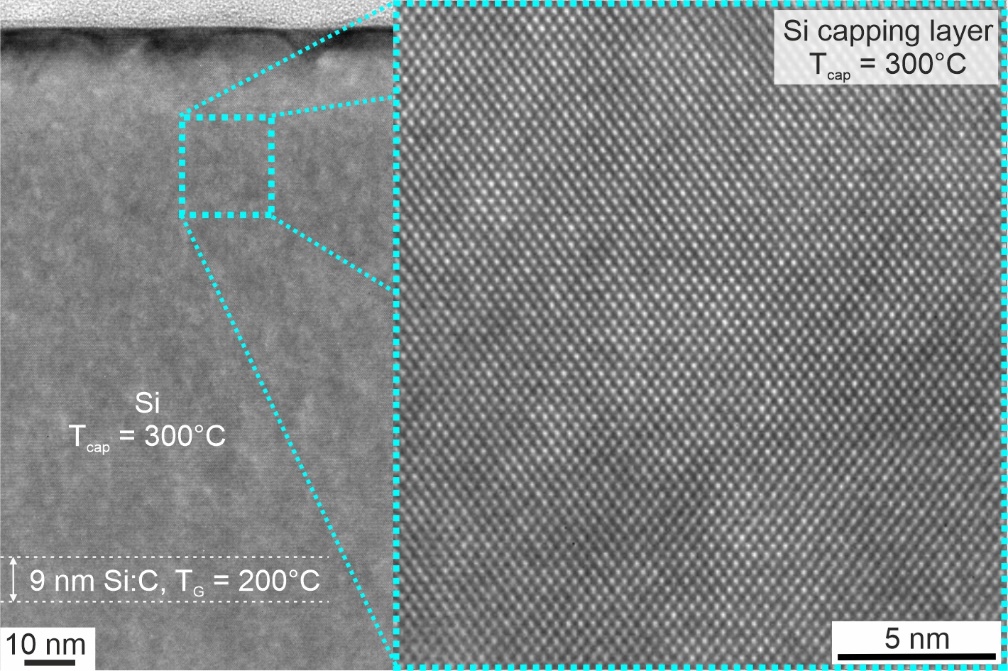


**Figure S2.** Cross-sectional transmission electron microscopy images of a 9 nm thick Si:C layer (C‑concentration of 3.8∙10^19^ cm^-3^) capped with crystalline Si grown at T_cap_ = 300°C.

**Comparison of the observed SiCC emitter type with traditional G-centers:**

In **Figure S3**a, we compare the PL emission spectrum of the investigated G′-centers to traditional G-centers fabricated via C-ion implantation into epitaxial Si layers. For this purpose, a 300 nm thick Si epilayer was grown on a full 4 inch FZ Si(001) wafer using a Si growth rate of 0.9 Å/s. During growth of the first 40 nm, T_G_ was linearly ramped from 475°C to 700°C while the remaining 260 nm were grown at a constant T_G_ = 700°C. After growth, the wafer has been annealed *in-situ* at 750°C for 1 hour. For the creation of high-density common G-centers, the wafer has been irradiated with 5∙10^14^ cm^-2^ ^12^C^+^ ions under a tilt angle of 7° using an ion energy of 34.5 keV. According to SRIM/TRIM simulations, the latter corresponds to a mean projected ion range of ~110 nm. The ion implantation was performed at room temperature w/o any post-implantation annealing. For the comparative PL measurements, 4 x 4 mm pieces have been cut from the wafer center followed by a (pre)cleaning procedure similar to that described in the methods section of the main article.

We observe the typical spectral fingerprint of the “traditional” G-center ensemble^7^, i.e. a bright zero-phonon line (ZPL) at ~1278.5 nm with an ensemble linewidth of 0.57 nm at FWHM, an extended phonon side band with a well-known local phonon mode(s) (LPM, also called E-line) at ~1381 nm (and ~1500.8 nm) and a Debye-Waller factor of up to 17.5% at 5 K. From the comparison (c.f. Figure S3a) with the G′ centers obtained/described/created in this work, the qualitative similarities between the PL spectra of the two emitter types become apparent, while all spectral features of the G′ emitters are shifted to longer wavelengths as compared to traditional G-centers.


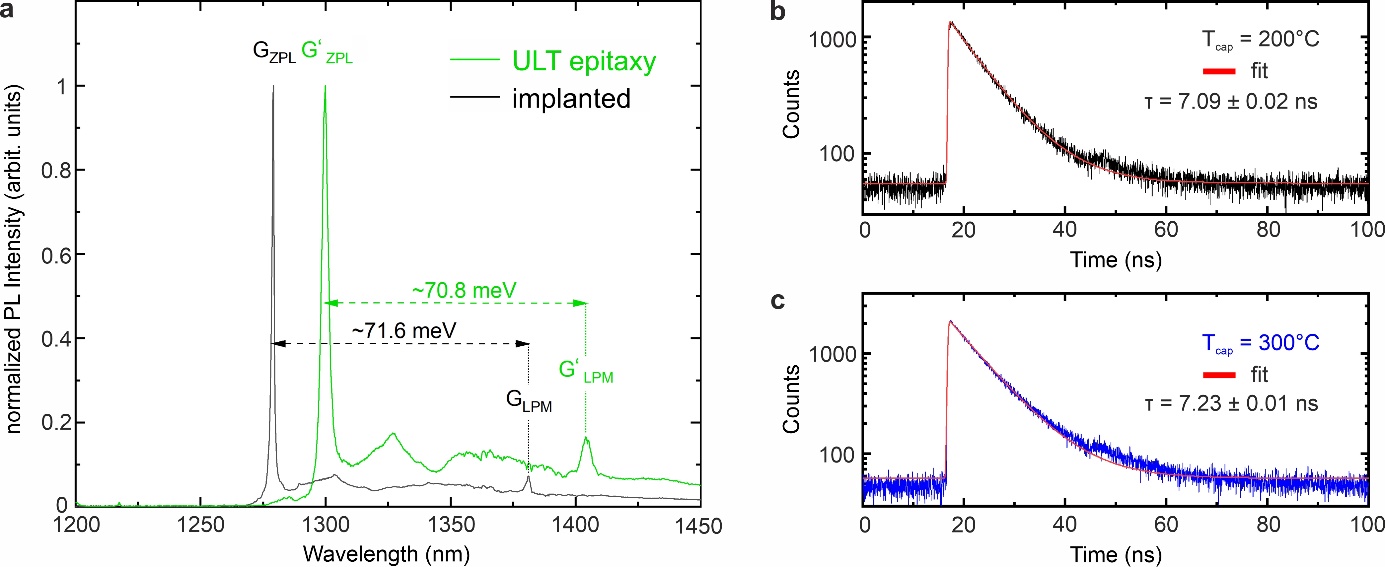


**Figure S3. a** Normalized PL spectra of G′ centers (green), observed after Si color center self-assembly with a high C-concentration of 3.8∙10^19^ cm^-3^, and the traditional G-centers (gray), observed after ^12^C^+^ ion implantation. The respective zero-phonon lines (G_ZPL_ and G′_ZPL_) as well as the local phonon modes (G_LPM_ and G′_LPM_) are indicated. **b, c** Distribution of the PL photon emission times of a G′-center into its ZPL after pulsed excitation by a diode laser emitting at 442 nm. From fitting the observed time distribution to a single exponential decay superimposed on a noise background, excited state lifetimes of about 7 ns are obtained for both the sample with a Si cap grown at **b** T_cap_ = 200°C and **c** 300°C.

**Lifetime measurements of G′-centers**

Figures S3b and S3c depict the results of excited state lifetime measurements of G′ emitter ensembles by time-correlated single photon counting (TCSPC) experiments. The respective samples consist of a 9 nm thick Si:C layer, doped to a C-concentration of 3.8∙10^19^ cm^-3^, and capped at two different capping layer growth temperatures, T_cap_ = 200°C (Figure S3b) and T_cap_ = 300°C (Fig. S3c). In both cases, the statistics of the time lag of ZPL photon emission after the excitation pulse were fitted with a single exponential function superimposed on a noise background, and the obtained excited state lifetimes amounted to τ ≈ 7 ns. For these measurements, the samples were excited by a pulsed single-mode diode laser (wavelength of 442 nm), with a pulse width of less than 200 ps at a repetition rate of 10 MHz and an average optical power ranging of 440 μW. To select the ZPL part of the resulting PL, the emission was spectrally filtered using a band-pass filter with a central wavelength of 1300 nm and a full-width-at-half-maximum of ~12 nm. The residual signal was further coupled to a single-mode fiber connected to a Single Quantum superconducting nanowire single photon detector (SNSPD) operated at 1.8 K. A PicoHarp 300 time tagging electronics operated in time-correlated photon counting (TCSPC) mode was then used to obtain the time-resolved PL decay.

***Ab initio* results**

We employed *ab initio* calculations to identify the G′-center and reveal its potential for quantum technologies. We assumed, based on the similarities between the features of the PL spectra of the G-center and G′-center, that the core of the defect is the atomic structure of the G-center but a nearby carbon impurity or Si self-interstitial may perturb it. The possible models are carbon substitutional (C_Si_), carbon interstitial (C_i_) or silicon interstitial (Si_i_) near the C_Si_-Si_i_-C_Si_ defect (so-called B-form of G-center that we simply label by ‘G’ for the sake of simplicity) that are depicted in **Figure S4**.


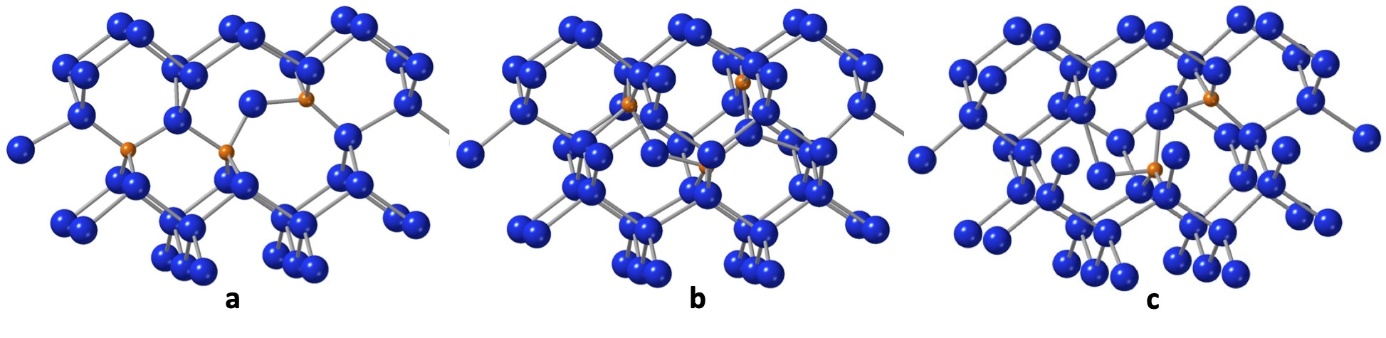


**Figure S4.** Considered models of the G′-center. **a** C_Si_+G, **b** C_i_+G and **c** Si_i_+G defects.

The electronic structure of C_i_+G and Si_i_+G defects exhibits additional in-gap defect levels when compared to that of G-center (c.f., **Figure S5**). On the other hand, the electronic structure of C_Si_+G defect is only a small perturbation to the G-center’s.


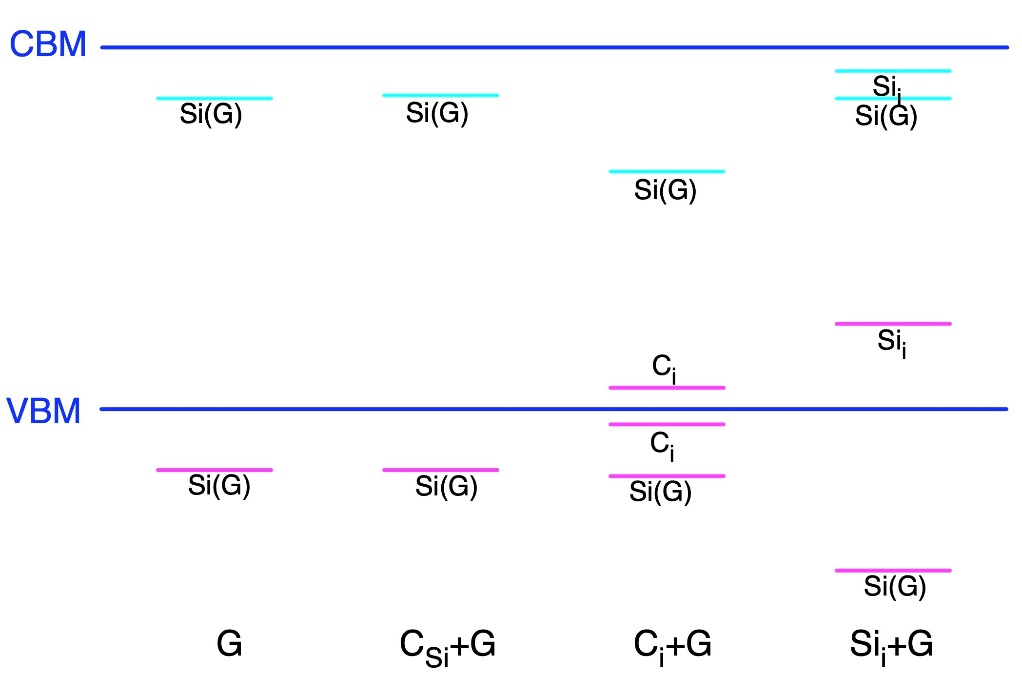


**Figure S5.** HSE06 Kohn-Sham levels of the states localized on defect atoms in the G-center and in the three models of the G′-center. The cyan and magenta-colored defect levels are empty and fully occupied, respectively. CBM: conduction band minimum. VBM: valence band maximum. The calculated band gap is 1.16 eV. Localization of these states are labeled as follows: Si(G): dangling bond of silicon interstitial in the C_Si_-Si_i_-C_Si_ unit; C_i_: dangling bond of carbon interstitial; Si_i_: dangling bond of silicon self-interstitial near the C_Si_-Si_i_-C_Si_ unit.

The calculated HSE06 ZPL energies are at 920 and 934 meV for C_Si_+G defect and G-center, respectively, so 14 meV red-shift appears when the ZPL of G-center is used as a reference. We note that we do not apply here local Hubbard-type correction to the p-orbital of Si_i_ in the C_Si_-Si_i_-C_Si_ unit in contrast to our previous work,^8^ because that correction might be defect specific that would prohibit the direct comparison of defects’ properties. In our experiment, the G′-center has 17 meV red-shift in the ZPL energy. The calculated local vibration mode associated with the C_Si_ motions in the C_Si_-Si_i_-C_Si_ chain (**Figure S6**a) that is a dominant peak in the phonon sideband of the PL spectra at 70.8 meV and 71.6 meV for the G′-center and G-center, respectively (Figure S3a) appears at 68.7 meV and 69.9 meV (Figure S6b) where the difference between these peaks in the experimental and simulated spectra is at 0.8 meV and 1.2 meV, respectively. The simulated PL phonon sideband agrees well with those in experiments where the prominent peak appears. Based on these results we assign G′-center to the C_Si_+G defect.


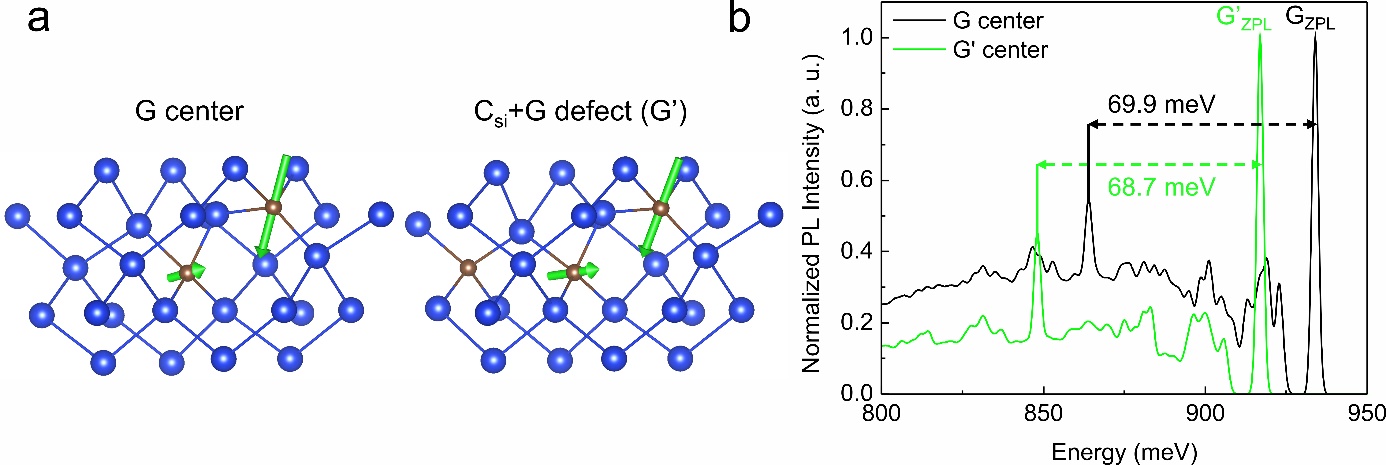


**Figure S6.** Prominent local vibration mode in the G-center and C_Si_+G defect (G′-center). **a** The geometry of the defects is depicted where the black circle indicates the location of C_Si_. The arrows show the direction and the amplitude of vibrating ions that are mainly localized on the two carbon atoms near the Si interstitial. **b** The simulated PL spectrum of the G-center and C_Si_+G defect (G′-center) with the prominent local vibration modes as depicted in **a**.

We also find that the G′-center has a triplet level at 0.63 eV above the ground state. The position of this metastable triplet level is reminiscent to the position of the metastable triplet level in the G-center (0.67 eV). In our previous work,^8^ we showed that this metastable triplet state was observed in the optically detected magnetic resonance (ODMR) of the G-center. Since the level structures of G-center (Ref. 8) and G′-center are very similar it is highly plausible that G′-center experiences the same ODMR effect. Although, the exact microscopic mechanism behind the ODMR effect for the G-center has not yet been revealed but the occurrence of the ODMR signal for the G-center clearly indicates that optical spin-polarization and optical readout of the electron spin in the metastable triplet state are doable. We propose that the same mechanism should occur in the G′-center too, so the same optical spin-polarization and readout techniques can be applied for the G′-center. We argue that our experiments realize a precise control of the location and density of the G′-center, which has become a very promising candidate for realizing spin-to-photon interface with emission in the telecom O-band.

**References:**

1. G. Davis, *The optical properties of luminescence centres in silicon*, Phys. Rep. **176**, 83 (1989); . https://doi.org/10.1016/0370-1573(89)90064-1
2. G. Davies, S. Hayama, L. Murin, R. Krause-Rehberg, V. Bondarenko, A. Sengupta, C. Davia, A. Karpenko, *Radiation damage in silicon exposed to high-energy protons*, Phys. Rev. B 73, 165202 (2006). https://journals.aps.org/prb/abstract/10.1103/PhysRevB.73.165202
3. Farrow, R. F. C., Ed. *Molecular Beam Epitaxy:  Applications to Key Materials*; Noyes Publications:  Park Ridge, NJ, 1995. ISBN-13: ‎ 978-0815513711
4. N. Yabumoto, *Analysis of molecular adsorbates on Si surfaces with thermal desorption spectroscopy*, AIP Conf. Proc. **449**, 696–701 (1998) https://doi.org/10.1063/1.56895
5. C. Porret, A. Hikavyy, J. F. Gomez Granados, S. Baudot, A. Vohra, B. Kunert, B. Douhard, J. Bogdanowicz, M. Schaekers, D. Kohen, J. Margetis, J. Tolle, L. P. B. Lima, A. Sammak, G. Scappucci, E. Rosseel, R. Langer, and R. Loo, *Very Low Temperature Epitaxy of Group-IV Semiconductors for Use in FinFET, Stacked Nanowires and Monolithic 3D Integration*, ECS Journal of Solid State Science and Technology **8**, P392-P399 (2019); https://iopscience.iop.org/article/10.1149/2.0071908jss
6. https://www.semiconductor-today.com/news_items/2023/jun/riber-intelliepi-150623.shtml
7. C. Beaufils, W. Redjem, E. Rousseau, V. Jacques, A. Yu. Kuznetsov, C. Raynaud, C. Voisin, A. Benali, T. Herzig, S. Pezzagna, J. Meijer, M. Abbarchi, G. Cassabois, *Optical properties of an ensemble of G-centers in silicon*, Phys. Rev. B **97**, 035303 (2018), https://link.aps.org/doi/10.1103/PhysRevB.97.035303
8. P. Udvarhelyi, B. Somogyi, G. Thiering, A. Gali, *Identification of a Telecom Wavelength Single Photon Emitter in Silicon*, Phys. Rev. Lett. **127**, 196402 (2021); . https://doi.org/10.1103/PhysRevLett.127.196402
